# Bioprocess systems engineering: transferring traditional process engineering principles to industrial biotechnology

**DOI:** 10.5936/csbj.201210022

**Published:** 2013-03-10

**Authors:** Michalis Koutinas, Alexandros Kiparissides, Efstratios N. Pistikopoulos, Athanasios Mantalaris

**Affiliations:** a Department of Environmental Science and Technology, Cyprus University of Technology, 95 Irinis Street, 3041, Limassol, Cyprus; b Centre for Process Systems Engineering, Department of Chemical Engineering, South Kensington Campus, Imperial College London, SW7 2AZ, London, United Kingdom

**Keywords:** Biological systems model development, Sensitivity analysis, Model analysis, Mechanistic model, Genetic circuit, Metabolic engineering

## Abstract

The complexity of the regulatory network and the interactions that occur in the intracellular environment of microorganisms highlight the importance in developing tractable mechanistic models of cellular functions and systematic approaches for modelling biological systems. To this end, the existing process systems engineering approaches can serve as a vehicle for understanding, integrating and designing biological systems and processes. Here, we review the application of a holistic approach for the development of mathematical models of biological systems, from the initial conception of the model to its final application in model-based control and optimisation. We also discuss the use of mechanistic models that account for gene regulation, in an attempt to advance the empirical expressions traditionally used to describe micro-organism growth kinetics, and we highlight current and future challenges in mathematical biology. The modelling research framework discussed herein could prove beneficial for the design of optimal bioprocesses, employing rational and feasible approaches towards the efficient production of chemicals and pharmaceuticals.

## Challenges in biotechnology

Biotechnology promises to deliver innovative and sustainable products and processes as solutions to various societal problems [1]. Important challenges in this field include sustainable production of biofuels from renewable sources as an alternative to fossil fuels, the exploitation of the metabolic capabilities of microorganisms towards the production of fine chemicals and pharmaceutical products, the construction of novel pathways for environmental bioremediation due to urban and industrial pollution, smooth transfer of lab scale experiments to pilot and production scale and the integration of biotechnology with chemical processes [2–4]. Recent advances in biotechnology have overcome several technological barriers encountered in industrial applications necessitating a deeper understanding of the underlying control mechanisms of cellular growth in order to achieve efficient and cost-effective bioprocesses.

Microorganisms hold great potential for industrial biotechnology, harbouring various metabolic pathways either for degradation of recalcitrant pollutants or the production of a series of compounds, which can be used as fuels, chemicals and pharmaceutical products [5]. However, the use of natural (wild type) microorganisms at large scale is usually hampered by sub-optimal bioprocesses in terms of yield, productivity and titre, along with the low tolerance of strains to process stresses, such as substrate and product toxicity, and fermentation inhibitors [6]. In order to improve the industrial efficacy of wild type microorganisms a variety of approaches have been proposed. Synthetic biology seeks optimal pathway configurations with the application of gene combinatorial methods to construct and consequently evaluate several metabolic pathways, combining genes from different sources. This *de novo* construction of artificial biological systems utilizes theoretical approaches for the design of modular system components [7–8]. Furthermore, systems biology methodologies attempt to use system-wide measurements obtained by high-throughput technologies in combination with mathematical methods for the elucidation and implementation of novel biosynthetic pathways and identification of genetic targets for modification [9]. Metabolic engineering also aims at the improvement of microbial strains for industrial application. Contrary to synthetic biology, metabolic engineering targets the optimisation of pathways by regulating the activity of intermediate reactions combining rational and combinatorial methods [10].

Mathematical models are increasingly becoming central to understanding and improving cellular based processes. However, with the field of biotechnology shifting from method development to application development [11], a systems biology approach of detailed, mechanistic modelling becomes problematic since modelling of complex biological systems inherently is an inverse problem that cannot be solved [12] and understanding of experimental information has lagged far behind data accumulation. Implementing microbial production on an industrial scale should focus towards bioprocess systems engineering strategies, which can ultimately enable control and optimisation at the bioprocess level [13].

## Challenges in biological modelling

Despite the economic turmoil of the last few years, Thomson&Reuters concur that the bio-industry is a viable platform for low risk investments with a good profit margin [14]. Nonetheless, the bio-chemical industry requires improved process efficiency; alas, the sophisticated mathematical toolset that led to the explosive growth of manufacturing capacity in traditional chemical industries, known as Process Systems Engineering (PSE), is not readily applicable to the bio-industry. Obstacles hindering the adaptation of traditional PSE approaches to bio-processing include the complexity of the biological systems, the limited understanding of the biological processes, and the resulting lack of adequate process models. In the absence of model-based approaches, process optimisation in the bio-industry relies on extensive, and in certain cases unnecessary, experimentation.

The use of model-based techniques can facilitate the reduction of unnecessary experimentation by indicating the most informative experiments and providing strategies to optimise and automate the process at hand, resulting in a cost and time reduction. Mathematical models of biological systems developed in the past integrate various degrees of structure and mathematical complexity. Models of single cells, cell populations and cell cultures have been utilized in understanding and improving biological systems, as well as in the optimisation and control of bioprocesses [13]. Indicatively, mathematical models have been applied to various extents in the design of optimal media [15], the identification of previously ignored growth limiting factors [16], the optimization of culture growth and productivity [17, 18], and in the application of control approaches to cell culture processes [19].

Yet when Pörtner and Schäfer [20] compared a selection of models for cell growth and metabolism of hybridoma cell lines through an analytic error and range of validity analysis, they found significant variations in the values of maximum growth rate, yield and Monod constants. They concluded that the model predictions involved significant errors, particularly due to the limited understanding of cellular metabolism and the narrow data ranges within which the models were valid. The observed discrepancies were partly attributed to the absence of a formalised approach for the proper identification of process parameters. It was concluded that specific process models should be used for the estimation of specific types of process parameters. For example, it was suggested that static batch cultures should be used for the determination of the maximum specific growth rate, but not for establishing a relationship between the growth rate and substrate concentration, whereas continuous cultures could yield reliable data due to the steady-state operation conditions. For very low substrate concentrations, they suggested using fed-batch cultures.

The large-scale generation of biological data obtained with a variety of high-throughput experimental technologies demand the development of integrated mathematical models of cellular processes [21]; alas, integration of mathematical modelling in bio-processing has proven to be challenging. The way biochemical engineers conceive and mathematically describe biological processes, by and large, is still defined by a mathematical formulation derived a century ago to describe enzyme kinetics [22]. Although the hypothetical system studied was the simplest possible, the conversion of one molecule of a given substrate to a product via a single enzymatic reaction, it has shaped the way we conceive kinetic rates in biology. Since then, the theory provided by Michaelis and Menten has evolved, being used as a starting point when attempting to describe much more complex systems, such as microbial growth [23]. Considering the developments in analytical and molecular biology over the past decades brings Bailey's [24] argument that the development of mathematically and computationally orientated research has failed to catch up with developments in biology. Mathematical biology today revolves around mathematical expressions developed a hundred years ago (Table 1).

**Table 1:** Enzyme and microbial growth kinetic expressions.

Name	Expression	Function
Michaelis-Menten	${\text{V}}_{0}=\frac{{\text{V}}_{\max}[\text{S}]}{{\text{K}}_{\text{m}}+[\text{S}]}$	Describes the kinetics of the simple enzyme catalysed reaction: $E+S\overset{k_{1}/k_{-1}}{↔}ES\overset{k_{2}}{\to }P$
Hill	$\theta =\frac{{\left[\text{L}\right]}^{\text{n}}}{{\left({\text{K}}_{\text{A}}\right)}^{\text{n}}+{\left[\text{L}\right]}^{\text{n}}}$	Describes the fraction of the macromolecule saturated by ligand as a function of the ligand concentration.
Monod	$\mu =\mu_{\max}\frac{\left[\text{S}\right]}{{\text{K}}_{\text{S}}+[\text{S}]}$	Describes microbial growth based on the consumption of one substrate.

*θ*: fraction of occupied ligand binding sites; *µ*: the specific growth rate of a microorganism; *µ*_*max*_: the maximum specific growth rate of a microorganism; *k*_*1*_: rate constant for association of substrate and enzyme; *k*_*-1*_: rate constant for dissociation of unconverted substrate from the enzyme; *k*_*2*_: rate constant for dissociation of product from the enzyme; *K*_*A*_: ligand concentration producing half occupation, which is also the microscopic dissociation constant; *K*_*m*_: Michaelis constant; *K*_*s*_: Monod coefficient; *[L]*: ligand concentration; *n*: Hill coefficient designating cooperativity; *[S]*: substrate concentration; *V*_*0*_: initial velocity of the enzymatic reaction; *V*_*max*_: maximum velocity of the enzymatic reaction.

Notable studies attempting to introduce a new approach to biological systems modelling include, but are not limited to, cybernetic modelling presented by Ramkrishna [25], the introduction of structure as defined by Fredrickson [26] and extended to the genetic level by Lee and Bailey [27, 28]. The concept behind cybernetic modelling is the adaptation of a mathematically simple description of a complex organism which is compensated for over-simplification by assigning an optimal control motive to its response [29]. Microbial cells growing in the presence of multiple substrates are assumed to follow an invariant strategy to optimise a certain goal by choosing which substrate to consume first. Thus, by assuming a multi substrate environment containing cells that follow different strategies of substrate consumption, those cells that choose to grow first on the fastest substrate available will grow much faster than cells that respond differently. After some time all the cells that remain in the environment will be those that have responded in the optimal manner.

Lee and Bailey [27, 28], extended the concept of structure to the level of nucleotide sequences. They introduced an explicit connection between a particular nucleotide sequence and the affinity of a particular protein for that sequence, which in turn influences the corresponding transcription event, deriving a quantitative mapping from nucleotide sequence to overall phenotype. Even though in his detailed review, Bailey [24], predicted that this new “genetically structured model” would be widely embraced in the future, supported by the advancement of the “omics” techniques, little work has been done in that direction.

Savageau [30–33] was amongst the first to investigate metabolic pathway control from a mathematical analysis point of view. A few years later the work of Kacser and Burns [34] and Heinrich and Rapoport [35] defined the field of Metabolic Control Analysis (MCA), which quantitatively studies the degree of flux control that is applied on a metabolic pathway by various effectors, such as enzyme activities and metabolite concentrations. Papoutsakis [36] demonstrated that it was possible to formulate balance equations using a metabolic map, a concept which later evolved into Flux Balance Analysis (FBA) [37, 38]. The idea of controlling flux balance through a given metabolic pathway towards achieving a desired overall behaviour (e.g. maximisation of product formation) was shaped into the principles of Metabolic Engineering developed by Bailey [39]. Stephanopoulos and co-workers [40–42] spearheaded the expansion of Metabolic Engineering which they defined as: “*the directed improvement of product formation or cellular properties through the modification of specific biochemical reaction(s) or the introduction of new one(s)*”.

Despite the significant efforts of the biochemical engineering community, the bio-industry has been slow to uptake and implement model-based approaches. Due to the lack of mechanistic information (and in many cases absence of proper modelling practice), mathematical models of biological processes are usually limited both in terms of range of validity and predictive capability. Model parameters are usually estimated without any *a priori* model analysis [43] and the reported values are only seldom accompanied by qualitative metrics (i.e. confidence intervals). This partly explains the significant deviation and inconsistency observed in the reported values of model parameters [20]. Moreover, mathematical models of biological systems, generally, lack transferability to other, even similar, processes without a complete re-estimation of the model parameters. Bioprocess models usually focus on the significant process variables and their interconnectivity around specific operating conditions. Furthermore, the primary “tools” utilised to describe the observed macroscopic behaviour are usually limited to nutrient and metabolite concentrations, a remnant from a period when those were the only readily measurable quantities analytically. If we imagine cellular metabolism as many pieces of rope tangled together, we are currently trying to untie the knot by pulling on one end of the rope alone. What is currently lacking is information from the other end of the rope, the genes, which are at the centre of control of cellular mechanism.

It comes as no surprise therefore, that model based optimisation of cell culture processes currently lags behind the developments in other process industries [17, 19]. Apart from the lack of robust and predictive process models, the lack of readily applicable on-line measurements of key process variables significantly hinders the application of traditional PSE tools to the bio-industry. The response of cell cultures to changes in the feeding strategy is usually monitored primarily through simple measurements such as the oxygen uptake rate and pH [16, 44–46]. The limited number of readily available on-line measurements in turn limits the complexity of the utilised model for deriving the optimal strategy. Thus, the common practice when estimating optimal feeding profiles is to base calculations on the cells’ need for the primary nutrients alone [17, 19, 44]. In an attempt to compensate for this simplification, excessive amounts of the primary nutrients are fed to the culture resulting in a net improvement of culture behaviour, even though this is known to be a sub-optimal approach [15].

Optimisation of cell culture processes largely depends on the balance between prolonged culture viability and increased productivity. Therefore, there is a need to identify and quantify this trade-off between growth and product formation in order to derive truly optimal culture conditions. Bailey [24] predicted the need to shift modelling focus upstream towards the genetic level where the kernel of the cell's control mechanism lies, in order to truly understand the dynamics of metabolism. In a nutshell, our research efforts should revolve around the following questions:

- Which genes are crucial for cellular growth and product formation? How do these genes interact between themselves and the environment?
- How does the process environment (indicatively: media composition, feeding schedule, osmotic pressure, mixing) affect the rate and yield of product formation?
- Can models describing basic phenomena (i.e. heterogeneity between cells [47]) make a significant impact on the improvement of prediction in industrial bioprocesses?
- How can an “off-line” model containing information from the genetic level be used to enhance the predictive capabilities of an unstructured model updated through on-line measurements?
- Can such a hybrid model be used for “***true***” bioprocess optimisation at both the bioreactor (through optimal operation) and the cellular (through appropriate genetic modifications) levels?

## Bioprocess systems engineering

**Figure 1:**
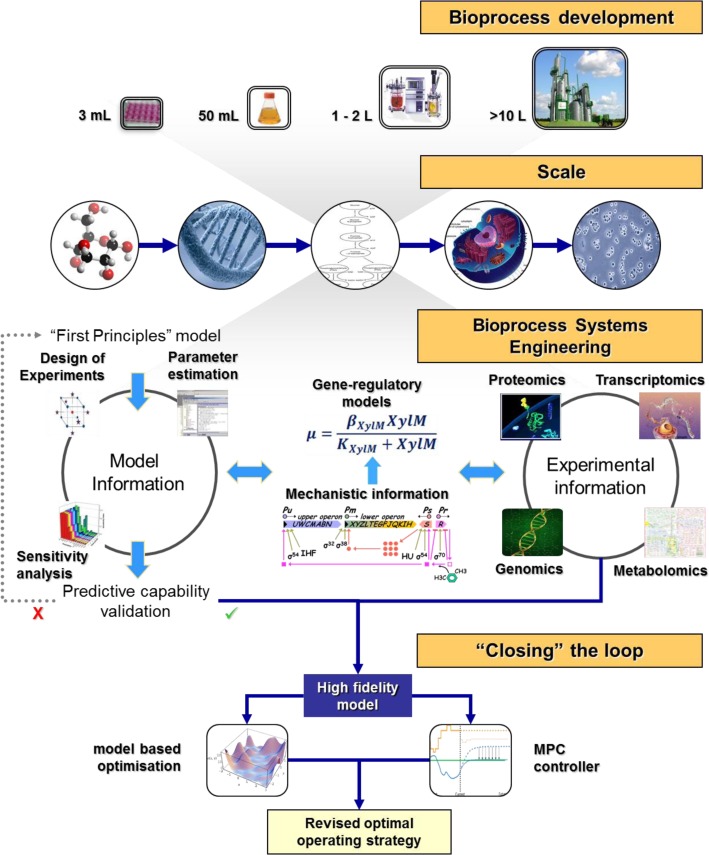
A *Bioprocess Systems Engineering* framework for model development in biological systems.

Present research efforts in systems biology are focused on the development of extremely complex models of intracellular pathways and the exploration of their qualitative characteristics. The problem with such detailed models is that it leads to a large number of model parameters, almost always larger than the number of measured experimental variables, which in turn limits the predictive capabilities of the model. In contrast, the use of overly simplified models may lead to the model's inability to capture experimental results. Thus, one of the challenges in the field is the development of high fidelity models able to capture the required biological functions while remaining computationally tractable. What is essentially lacking is a formalized structure to guide the development of tractable yet still realistic mathematical models whilst maximizing the information content of the experimental measurements. Such a systematic approach to modeling biological systems has been shaped by the work of [48–53] and is depicted in Figure 1. Each step of the framework organizes and directs the flow of experimental information in an effort to alleviate uncertainty where possible. More specifically:

*Step 1. Model development:* One of the challenges is the development of a model of adequate fidelity in order to capture the required biological functions while remaining computationally tractable. However, high fidelity models inherently contain a large number of parameters. It is important to define the aim of the model *a priori* and choose an appropriate model type (i.e. structured vs. unstructured) depending on both the scope and the number of available experimental measurements. For example, during a high-throughput screening test a small, easily tuneable model able to capture basic metabolic characteristics would be preferred in order to assist in the identification of high-capacity cell lines. However, when designing new cell lines by inserting mutations, that could potentially increase product yield, a detailed model would be essential. Model equations are defined through ‘first principles’ relationships. Let us name the developed mathematical model as $g(x,\bar{P})$ where x denotes the input vector, and ${\bar{P}}_{I}$ (where i = 1,…,ν) denotes the parameter vector.

*Step 2. Sensitivity analysis* [50, 53] *:* Investigates how the uncertainty introduced through the parameter value estimates affects the model's output and defines parameters crucial to the model's output. Model parameters with low sensitivities can be set to their nominal values. The highly non-linear nature of models of biological systems favours the use of global methods (for example Sobol’ global indices). It has been shown that sensitivity indices change along the time trajectories of the studied model outputs [50]; therefore it is advisable to conduct global sensitivity analysis (GSA) at various time points as this provides valuable information for the design of tailor made experiments.

The output of SA will be a vector of size ν, containing the sensitivity indices (SI) of the model parameters. Consequently an empirical threshold criterion, determined by the modeller, is applied in order to discriminate the significant from the insignificant model parameters. Any parameters with values below the set threshold are considered insignificant to the model output and are allocated in a partition of the parameter vector termed ${\bar{p}}_{j}^{1}$ (j = 1,…,ν’). The remaining parameters whose SI is above the threshold value are allocated in a second partition of the parameter vector termed ${\bar{p}}_{k}^{2}$ (k = 1,…,ν’’). The sum of ν’ and ν’’ should equal the size of the parameter vector ${\bar{P}}_{I}$, at all times. The values of the parameters in partition ${\bar{p}}_{j}^{1}$ are set to the nominal values, which can be derived either from existing literature or from a parameter estimation algorithm, hence yielding the parameter vector ${\bar{p}}_{j}^{nom}$.

*Step 3. Optimal experimental design (DoE)* [52, 56] *:* Based on the available experimental measurements, the aim of Model-Based Experiment Design is to design experiments that maximize the information content of the measurements in the context of estimating the significant model parameters [52, 53]. This is equivalent to minimizing the variances of the parameters to be estimated. DoE aims to address the following questions:

- What should be the initial conditions for the experiment?
- How long should we run the experiment for?
- How should we vary the controls (e.g. the time profiles of feed flow rates)?
- When should we take the measurement samples?

Based on an optimality criterion (for example D-optimality) experiments are specifically designed for the determination of the significant parameters (vector ${\bar{p}}_{k}^{2}$) and once the experimental data is available the values for parameters ${\bar{p}}_{k}^{2}$ are determined explicitly, yielding vector ${\bar{p}}_{k}^{\exp}$.

*Step 4. Range of validity* [52] *:* The predictive capability of the refined model needs to be tested against a set of independent experiments with varying environmental conditions. If the model is not able to describe the experimental trends satisfactorily then a new model of increased fidelity needs be developed and the whole process needs to be repeated.

*Step 5. Model based optimisation* [57] *:* Dynamic optimization techniques can be used to identify worst- and best-case scenarios for the operation of fed-batch and continuous cell cultures. The former should be avoided and the latter needs to be further refined experimentally so as to reach truly optimal operating conditions. Of particular interest are dynamic optimisation techniques that can remain computationally inexpensive whilst dealing with model uncertainty and scarce sampling times.

The use of model-based techniques can facilitate the reduction of unnecessary experimentation by indicating the most informative experiments and providing strategies to optimise and automate the process at hand. The presented research approach attempts to integrate modelling, experimental design and validation with model based control and optimisation within a closed loop framework, that leads to increased productivity and reduced production costs for cell culture systems. The integration of these four research tools represents an elegant interdisciplinary approach that addresses the complicated research and industrial problem of model-based control and optimisation of cell culture processes.

Traditional models of microbial growth kinetics are based on the assumption that description of the rate-limiting step produces an adequate description of the process. Therefore, the Monod model, which is perhaps the best classical description of growth kinetics, is based on the assumption that culture growth is limited by a single rate-limiting enzyme reaction following the well-known Michaelis-Menten kinetics [23]. However, although traditional models can be in some cases very accurate, they are apparently not capable of capturing the regulatory effects controlling upstream the production of catabolic enzymes, providing a rather simplified and idealised view of complex biological processes [58]. The current progress in molecular biology can be used to unravel the underlying biological mechanisms that regulate gene expression and cellular function. High-throughput experimental technologies are able to elucidate the behaviour of a biological system at a holistic level. The results generated are known as ‘omics’ data and constitute of genomics, trascriptomics, proteomics and metabolomics, which measure gene, transcript, protein and metabolite profiles of cells [59]. During the past few years the advances in the ‘omics’ technologies have facilitated better understanding of the function of microorganisms as industrial “cell-factories”. This recent ability to acquire mechanistic knowledge of cell function at local and global level enables the replacement of empirical models with mechanistic ones, thus advancing the development of efficient bioprocess models for industrial biotechnology [60].

Various mathematical modelling approaches have been applied to study the properties of biological systems. FBA has been employed for analysing the properties of large metabolic networks and predicting the phenotypic behaviour of microorganisms [61]. Sorting of large amounts of biological data can be also done with the use of Boolean models, which make the assumption that gene expression is discrete [62]. On the contrary, when the study of smaller systems is required, dynamic analysis employing a set of ordinary differential equations (ODEs) can be applied to describe cellular behaviour in a mechanistic way, providing information about the kinetics of molecular interactions [63]. Moreover, the stochastic kinetics modelling framework considering the stochastic nature of biochemical reactions has been used to predict the concentration of molecular components in the cell [64]. The recent effort to build a whole-cell model has made the development of integrative modelling approaches necessary for the analysis of metabolism. Therefore, large-scale metabolic (FBA) and regulatory network (Boolean) models have been used as a scaffold with which ODE-based models can be integrated to study detailed models of sub-cellular networks in the context of their global effects [65]. Most of the integration efforts of large-scale regulatory and metabolic dynamic models assume steady state for the fast reactions of the metabolism, while slow reactions occasionally change the key parameters (i.e., metabolic fluxes) for the fast reactions [66]. A valuable approach that complements previous studies demonstrates that focusing on the development of validated mechanistic models of key genetic circuits, describing the molecular and genetic events that control the synthesis of enzymes, can significantly improve the prediction of bioprocess performance. This approach was initially established with the development of mechanistic models of circuits validated at the enzymatic level. Kremling *et al* [67] constructed a very detailed model describing the dynamics of gene regulation in the famous *lac* operon, producing an accurate description of glucose and lactose uptake and metabolism. In line with the above, the development of a simple dynamic gene regulation model to describe biomass and penicillin production in a chemostat, which was validated using only enzyme activity assays, has been recently applied for the prediction of bioprocess performance [68].

**Figure 2:**
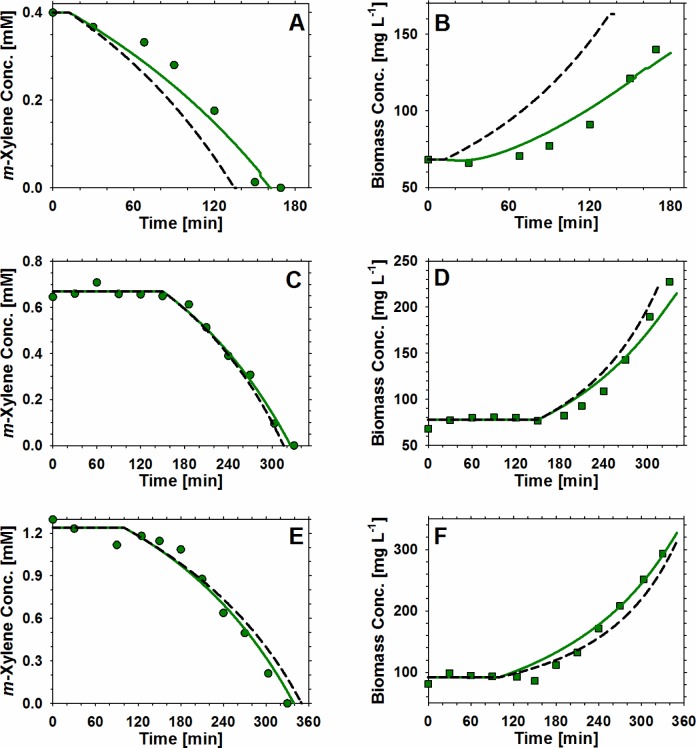
**Comparisons of *Pseudomonas putida* mt-2 growth kinetics predictions for mechanistic and Monod-type models**. The TOL model was used to calculate the concentration of mRNA and rate-limiting enzymes regulating *m*-xylene biodegradation and biomass growth respectively. The concentration of each enzyme was used to predict *m*-xylene and biomass dynamic profiles in the combined model, which is compared to the Monod model. Shown are simulation and experimental results for three predictive experiments at various initial *m*-xylene concentrations. (**A-B**) 0.4 mM *m*-xylene, (**C-D**) 0.7 mM *m*-xylene, and (**E-F**) 1.3 mM *m*-xylene. 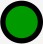: *m*-xylene concentration - experimental; 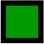: biomass concentration - experimental; 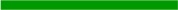: combined model; 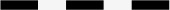: Monod model. For more details see [69].

We have previously demonstrated that the development of validated mechanistic models of key genetic circuits, describing the molecular and genetic events that control the synthesis of enzymes, can significantly improve the prediction of bioprocess performance. Specifically, we have developed a novel mathematical model describing the function of the TOL (pWW0) plasmid of *Pseudomonas putida* mt-2 [69], which is considered a paradigm of specific and global regulation, encoding the enzymes for degradation of major environmental pollutants. The validated model of the TOL plasmid computed the relative mRNA transcript levels of the encoded genes and the amount of the translated rate-limiting enzymes regulating *m*-xylene biodegradation and biomass growth respectively. The concentration of rate-limiting enzymes were subsequently linked to specific growth and substrate utilisation rates, effectively describing the dynamics of the system, at both the gene expression (regulation) and macroscopic (kinetics) levels. The concentration of each enzyme was used to predict *m*-xylene and biomass dynamic profiles in the combined model, which was compared to the Monod model [23]. Equations 1 and 2 demonstrate that the rate-limiting enzyme concentration and the specific growth rate depend on mRNA levels and as shown on Figure 2, the mechanistic model developed was substantially more accurate than the Monod model for a wide range of initial *m*-xylene concentrations in batch cultures. This study demonstrates that linking the functional relationships between genetic circuit components is not only able to predict specific cellular functions, but also to provide the opportunity to shift from unstructured and empirical models to advanced mechanistic models accurately predicting bioprocess performance.

$$\frac{dXylM}{dt}=\beta_{XylM}Pm_{TC}-\alpha_{XylM}XylM$$

$$\mu =\frac{\beta_{XylM,b}XylM}{K_{XylM,b}+XylM}$$

*α*_*XylM*_: XylM degradation and dilution due to cellular volume increase;*β*_*XylM*_: the translation rate based on *Pm* promoter driven mRNA synthesis; *β*_*XylM,b*_: the maximum specific growth rate of biomass based on XylM; *µ*: the specific growth rate; *K*_*XylM*_: the saturation constant for XylM; *Pm*_*TC*_: relative mRNA concentration from *Pm* promoter; *t*: time; *XylM*: the concentration of the assumed rate-limiting enzyme of the *meta* pathway encoded in TOL.

The development of mechanistic models that utilize biological information obtained by advanced experimental techniques is currently enriching our understanding of bioprocesses, assisting the transition from empirical to detailed kinetic models [60]. This approach is realistic due to the progress in sequencing and genetic engineering, which have made the study of wild-type genetic circuits with mathematical models a feasible endeavour [70]. Various studies presented in the past few years have focused on dynamic modelling of genetic circuits [71–75]. As the function of a greater portion of the gene control network is clarified, it will be possible to apply mechanistic mathematical models that describe the dynamics in key regulatory systems for the design of optimal bioprocesses.

## Future opportunities

As discussed above, several mathematical models of key genetic circuits are currently available for a variety of bioprocesses with the level of fidelity that would be required for industrial applications. However, mechanistic models are still not yet substantially applied for industrial bioprocess development [60]. Nevertheless, interest in them has grown considerably as mechanistic models may provide an outstanding summary of process knowledge [76]. The improvement of experimental techniques and computational power provide the opportunity to develop increasingly complex models. Given these advances, mechanistic models are expected to be gradually used to a greater extent in the future and may eventually become routine for industrial application. Consequently, the construction of complex models that capture the dynamics of various interacting genes, proteins and metabolites will be used to simulate conditions that are too expensive or time-consuming to be tested experimentally intervening beneficially in bioprocess development [77].

Many of the candidate strains for application at large scale are capable of producing very small quantities of the desired product and under conditions other than those usually applied in the industry [78]. The improvement of those microorganisms remains one of the greatest challenges in biotechnology and requires the combination of engineering and molecular biology disciplines for the analysis and modification of strains and bioprocesses. The development of next generation bioprocesses, with high selectivity for the production of pharmaceuticals and for production of chemicals from renewable sources is an effort where process systems engineers are expected to have an important role [79]. In line with the above, the modelling framework discussed in the present work provides a methodology that organises experimental information systematically, omitting unnecessary experiments and developing models with *a priori* established aims. Future studies may also utilize the current progress in molecular biology to construct detailed mechanistic models of key regulatory processes facilitating the development of high fidelity bioprocess models. Maybe then we can upgrade the simplified models that describe microbial growth kinetics based on enzyme kinetics with more detailed mechanistic models that capture the dynamics of gene regulation controlling upstream the production of catabolic enzymes.
